# Coping, stress, and burden among caregivers of older adults with Alzheimer's: a network analysis

**DOI:** 10.1590/1980-5764-DN-2025-0367

**Published:** 2025-12-05

**Authors:** Izabela Vitória Pereira Marques, Chia Chen Lin, Eduardo Quadros da Silva, Bruno Fernando de Souza Tavares, Talita Cezareti da Silva, José Roberto Andrade do Nascimento, Daniel Vicentini de Oliveira

**Affiliations:** 1 Universidade Cesumar, Departamento de Pós-Graduação em Promoção da Saúde, Maringá PR, Brazil.; 2 Universidade Cesumar, Departamento de Graduação em Fisioterapia, Maringá PR, Brazil.; 3 Instituto Nacional de Cardiologia, Departamento de Pós-Graduação em Ciências Cardiovasculares, Rio de Janeiro RJ, Brazil.; 4 Universidade da Força Aérea, Programa de Pós-Graduação em Desempenho Humano Operacional, Rio de Janeiro RJ, Brazil.; 5 Universidade Estadual de Maringá, Departamento de Ciências do Movimento Humano, Ivaiporã PR, Brazil.

**Keywords:** Aging, Dementia, Home Care Services, Subjective Stress, Envelhecimento, Demência, Serviços de Assistência Domiciliar, Estresse Subjetivo

## Abstract

Caregiving for older adults with Alzheimer's disease (AD) has intensified in Brazil, highlighting the emotional and physical burden faced by caregivers. Understanding the factors contributing to this burden is essential for developing more effective and sustainable support strategies.

**Objective:**

The aim of this study was to analyze the relationship between coping strategies, caregiver burden, and perceived stress using network analysis.

**Methods:**

This was a quantitative, analytical, observational, and cross-sectional study with a final sample of 126 formal and informal caregivers from different regions of Brazil. Data were collected via an online questionnaire, using the Perceived Stress Scale, Zarit Burden Interview, and the Coping Strategies Inventory (EMEP). Statistical analysis was conducted with JASP software. Data showed non-parametric distribution, and Spearman's correlation and LASSO network analysis were applied.

**Results:**

Results revealed weak but significant positive correlations (p<0.05; r<0.40) among coping domains. Problem-focused coping was negatively correlated with burden (r=-0.34) and stress (r=-0.60), while emotion-focused coping showed positive correlations with both (r=0.40; r=0.42). Religious/fantasy coping and seeking social support were also positively associated with burden. The LASSO network highlighted emotion-focused and religious coping as central nodes. Problem-focused coping was negatively associated with stress, while emotional and religious strategies were linked to increased burden and stress. Centrality indices indicated emotional coping and religious practices as highly influential.

**Conclusion:**

These findings underscore the protective role of problem-focused coping and the need for targeted interventions to foster adaptive strategies and support caregiver well-being.

## RESUMO.

O cuidado de idosos com doença de Alzheimer tem se intensificado no Brasil, revelando a sobrecarga emocional e física enfrentada pelos cuidadores.

**Objetivo:**

Analisar a relação entre estratégias de enfrentamento, sobrecarga do cuidador e estresse percebido utilizando análise de redes.

**Métodos:**

Estudo transversal, com amostra final de 126 cuidadores formais e informais de diferentes regiões do Brasil. Os dados foram coletados por meio da Escala de Estresse Percebido, da Escala de Sobrecarga de Zarit e do Inventário de Estratégias de Enfrentamento. A análise estatística foi realizada pela correlação de Spearman e a análise de redes LASSO.

**Resultados:**

O enfrentamento focado no problema apresentou correlação negativa com a sobrecarga (r=-0,34) e com o estresse (r=-0,60), enquanto o enfrentamento focado na emoção apresentou correlação positiva com ambos (r=0,40; r=0,42). As práticas religiosas/fantasiosas e a busca por suporte social também se associaram positivamente à sobrecarga. A análise de rede LASSO destacou o enfrentamento focado na emoção e o enfrentamento religioso como nós centrais. O enfrentamento focado no problema esteve negativamente associado ao estresse, enquanto as estratégias emocionais e religiosas se relacionaram ao aumento da sobrecarga e do estresse. Os índices de centralidade indicaram o enfrentamento emocional e as práticas religiosas como altamente influentes.

**Conclusão:**

Os achados reforçam o papel protetivo do enfrentamento focado no problema e a necessidade de intervenções direcionadas para promover estratégias adaptativas e apoiar o bem-estar dos cuidadores.

## INTRODUCTION

Alzheimer's disease (AD) affects around 50 million people globally, including approximately 1.2 million in Brazil, with numbers expected to grow due to population aging and increased life expectancy^1^. It is a progressive, incurable neurodegenerative disease, marked by cognitive impairments, behavioral and personality changes, and motor deficits — such as loss of strength, coordination, and gait — resulting in increasing dependence for activities of daily living^2^. Anatomical and physiological changes may be detected even before clinical symptoms emerge^2^.

AD evolves through three main stages—early, intermediate, and advanced — accompanied by increasing caregiver involvement^3^. Caregivers can be formal (non-relatives) or informal (typically family members). In Brazil, most caregivers are informal, mainly women with low income and limited education, who themselves often have chronic diseases^3,4^. This context amplifies the physical and emotional toll of caregiving, making it essential to understand the coping strategies adopted^5^.

Coping mechanisms serve to manage daily stress and overload. While formal caregivers tend to rely on leisure activities, informal caregivers often find meaning and relief in faith and religiosity^6,7^. However, continuous caregiving without adequate support frequently leads to neglect of their own health, fatigue, psychological distress, and reduced quality of life^8^. Studies by Tedrus et al.^9^ and Lemos et al.^10^ confirm that faith and spirituality are the most reported strategies among caregivers, functioning as emotional support^11^.

Despite advances in understanding the quality of life of people with AD^12,13^, few studies focus on caregivers’ physical and psychological health. Caregiver burden, social exclusion, lack of health education, and absence of targeted public policies remain pressing issues^14^. Evidence suggests that effective coping can reduce stress and improve quality of life^15^, yet more investigation is needed to guide interventions^16^. Therefore, this study aimed to analyze the relationships between coping strategies, caregiver burden, and perceived stress among caregivers of older adults with AD, using network analysis.

## METHODS

This quantitative, analytical, observational, and cross-sectional study was approved by the Research Ethics Committee (CEP) of Cesumar University, under approval number 6.001.701/2023. The study followed the Strengthening the Reporting of Observational Studies in Epidemiology (STROBE) guidelines.

### Participants

The sample consisted of formal (professional) and informal (family) caregivers of older adults with AD, residing in different regions of Brazil. Participation was voluntary through the dissemination of a link for online data collection.

- Inclusion criteria were as follows:
- Being a formal (professional) or informal (family) caregiver of an older adult with a self-reported medical diagnosis of AD;
- Being aged 18 years or older;
- Caregivers of both sexes;
- Residing in Brazil;
- Providing care to older adults living in the community (not institutionalized or hospitalized); and
- Agreeing to participate by accepting the online informed consent form.
- Exclusion criteria were as follows:
- Caregivers of institutionalized or hospitalized older adults;
- Incomplete responses to the online questionnaire;
- Respondents younger than 18 years; and
- Individuals who reported not being caregivers of older adults with AD.

As there is no exact or estimated number of caregivers of older adults with AD in Brazil, and data collection was conducted online, the sample was non-probabilistic and based on convenience. Therefore, no a priori sample size calculation was possible. However, the final sample of 126 caregivers meets the criteria established in the literature for exploratory studies, which recommend samples with more than 100 respondents to ensure adequate descriptive and inferential statistical analysis^17^.

### Instruments

A questionnaire developed by the authors was used to assess the sociodemographic, health, and care profiles of older adults with AD. It included questions about age, age group, sex, family income, education level, retirement status, medication use, associated diseases (comorbidities), and time since AD diagnosis.

A separate questionnaire was used to assess caregiver characteristics, covering variables such as age, age group, sex, family income, education level, medication use, presence of chronic conditions, duration of caregiving, daily hours dedicated to care, and whether the caregiver lived with the older adult with AD.

Perceived stress was assessed using the Perceived Stress Scale (PSS), which consists of 14 items with response options ranging from 0 to 4 (0=never, 1=rarely, 2=sometimes, 3=fairly often, 4=very often). Positively worded items (items 4, 5, 6, 7, 9, 10, and 13) were reverse-scored (0=4, 1=3, 2=2, 3=1, 4=0), while negatively worded items were scored directly. The total score ranges from 0 to 56, with higher scores indicating higher levels of perceived stress^18^.

The Zarit Caregiver Burden Interview (ZBI) was used to evaluate caregiver burden. This instrument assesses caregivers’ perceptions of how caregiving affects their personal, social, financial, physical, and mental health. It consists of 22 items rated on a 5-point Likert scale: 0 (never), 1 (rarely), 2 (sometimes), 3 (quite frequently), and 4 (nearly always). The total score ranges from 0 to 88, with higher scores indicating greater caregiver burden^19^.

Coping strategies were assessed using the Ways of Coping Scale (EMEP), identifying how individuals respond to stressful situations — in this case, caregiving for a person with AD. It is a 45-item Likert-type questionnaire with five response options: 1=I never do this, 2=I rarely do this, 3=I sometimes do this, 4=I often do this, and 5=I always do this^20^.

Although total scores can range from 45 to 225, interpretation focuses on the specific scores of four coping factors: problem-focused coping, emotion-focused coping, seeking social support, and religiosity/fantasy thinking. Higher scores in problem-focused coping and seeking social support indicate more adaptive strategies and better psychological adjustment. Conversely, higher scores in emotion-focused or religious/fantasy-based coping may reflect a less effective strategy, potentially indicating avoidance or difficulty in actively dealing with the stressor, depending on the context^20^.

### Data collection procedures

The study followed the ethical standards for human research outlined in Resolution 466/2012 of the Brazilian National Health Council. Data were collected using an online form hosted on the SurveyMonkey platform. Participants who agreed to take part in the study first read and accepted the informed consent form (ICF) by selecting "I agree" on the online form.

The survey link, which hosted all the study questionnaires, was disseminated through the authors’ social media platforms (Facebook™, Instagram™, and WhatsApp™).

The questionnaire remained available for responses for 90 days. Before completing the survey, participants received brief instructions, including information about the study's objective, target population, and estimated time for completion (approximately 15 min).

### Data analysis

Data were analyzed using descriptive and inferential statistics in JASP software. The normality of the data was tested using the Kolmogorov-Smirnov test, revealing a non-parametric univariate distribution. Spearman's correlation was used to assess relationships between variables. Values were considered statistically significant when p<0.05.

A network analysis technique investigated the complex interaction between study variables. Specifically, a Least Absolute Shrinkage and Selection Operator (LASSO) network was generated to calculate partial correlations between all variables, while controlling for the influence of others. The LASSO method reduces trivially small correlations to zero, thus producing a more interpretable network by removing potentially spurious associations^21^.

In this network, "nodes" (circles) represent variables and "edges" connect them. The color of the edge indicates the direction of the relationship (blue for positive, red for negative), and the thickness represents the strength of the association. Their calculated associations determine node placement within the network^22^.

Additionally, the following centrality indices were used to identify the most influential nodes: Strength of connections; Closeness, which measures the average distance from one node to all others and indicates how easily information can travel from that node through the network; Betweenness, which measures how often a node lies on the shortest path between other nodes, reflecting its potential to influence other variables; Expected influence estimates a node's importance in activating or deactivating other nodes in networks that include negative edges^23^.

## RESULTS

Notably, 126 caregivers participated in the study, including 118 females and 8 males, aged between 22 and 80 years (M = 51.36; SD = 10.90). The majority of caregivers were between 40 and 59 years old (61.9%), had a partner (54.8%), held a higher education degree (60.3%), identified as white (64.2%), and reported a monthly income of one to two minimum wages (42.1%).

Table 1 presents the descriptive statistics and correlations between coping strategies, stress, and caregiver burden. Overall, caregivers reported a moderate level of perceived stress (M=29.20; SD=10.05). Among the coping strategies, the highest scores were observed for problem-focused coping (M=64.59; SD=11.31), followed by emotion-focused coping (M=40.35; SD=6.31), religious/fantasy-based coping (M=23.85; SD=4.60), and seeking social support (M=16.48; SD=3.47). Finally, caregivers reported a slight to moderate level of burden (M=59.07; SD=15.83).

**Table 1:** Descriptive statistics and correlations between coping strategy domains, stress, and caregiver burden levels. Brazil, 2023.

Variables	1	2	3	4	5	6
1. Problem-focused coping	-	0.17*	0.20*	0.13	-0.60†	-0.34†
2. Emotion-focused coping		-	0.36†	0.26†	0.42†	0.40†
3. Religious/fantasy-based coping			-	0.36†	0.05	0.25†
4. Seeking social support				-	-0.03	0.17*
5. Perceived stress					-	0.61†
6. Caregiver burden level						-
Mean	64.59	40.35	23.85	16.48	29.20	59.07
Standard deviation	11.31	6.31	4.60	3.47	10.05	15.83

Notes: Spearman's correlation.

* Correlation is significant at the 0.01 level (2-tailed);

† Correlation is significant at the 0.05 level (2-tailed).

The analysis of the correlation matrix for the investigated variables (Table 1) revealed positive, significant (p<0.05), and weak correlations (r<0.40) among the domains of coping strategies. Problem-focused coping showed negative correlations of weak to moderate strength (r between −0.34 and −0.60) with caregiver burden and perceived stress, respectively.

Emotion-focused coping was moderately and positively correlated with stress (r=0.42) and burden (r=0.40). Meanwhile, religious/fantasy-based coping and seeking social support were also positively correlated with burden, though with weak intensity (r=0.25 and r=0.17, respectively). Finally, perceived stress was moderately and positively associated with caregiver burden (r=0.61).

**Figure 1:**
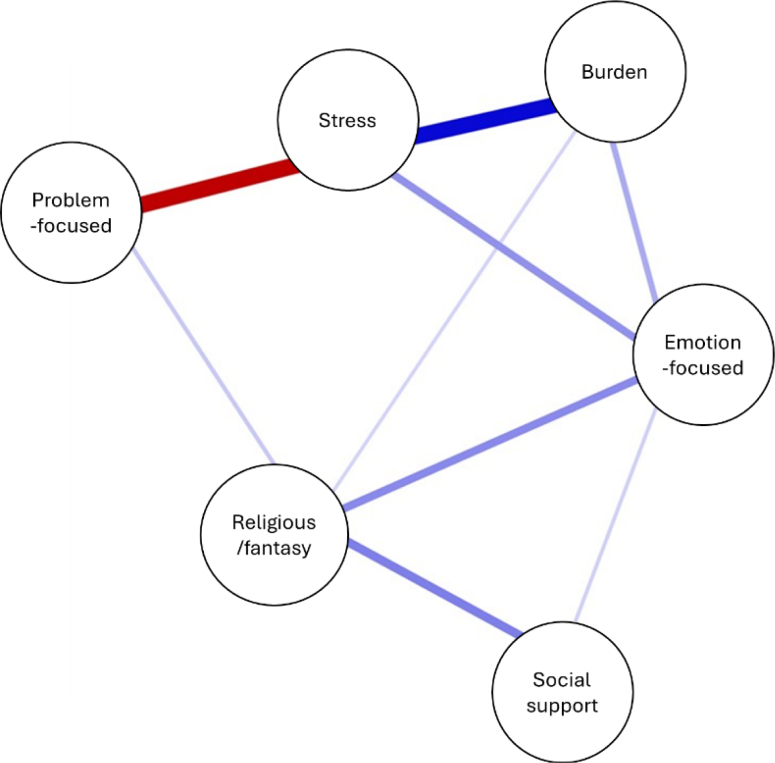
Network structure (n=126) of coping strategy domains, stress, and burden level. Brazil, 2023.

The resulting LASSO network (Figure 1) revealed an apparent clustering and separation of variables, with coping strategy domains positively associated, indicating mutual interaction among these variables. The network also highlighted a negative association between problem-focused coping and perceived stress, reinforcing the protective role of this strategy.

Figure 1 highlights that emotion-focused coping and religious/fantasy-based coping were positioned at the center of the network, establishing the main positive connections with stress and caregiver burden. It is noteworthy that problem-focused coping showed a negative connection with stress, while emotion-focused coping had a positive connection. Additionally, emotion-focused and religious/fantasy-based coping were negatively associated with caregiver burden. Finally, stress and caregiver burden were positively connected within the network.

Table 2 presents the weights of all associations within the network. A closer examination of the interaction between coping strategies and stress/burden reveals that problem-focused coping was the most relevant indicator of lower stress levels (r=-0.41). On the other hand, emotion-focused coping emerged as a contributing factor to increased caregiver burden and stress (r=0.14 and r=0.18, respectively), while religious/fantasy-based coping showed a weak positive association with caregiver burden (r=0.07).

**Table 2:** Weight of the associations within the network analysis. Brazil, 2023.

Variables	1	2	3	4	5	6
1. Problem-focused coping	-					
2. Emotion-focused coping		-				
3. Religious/fantasy-based practices	0.09	0.19	-			
4. Seeking social support		0.08	0.21	-		
5. Stress	-0.41	0.18			-	
6. Burden level		0.14	0.07		0.40	-

**Figure 2:**
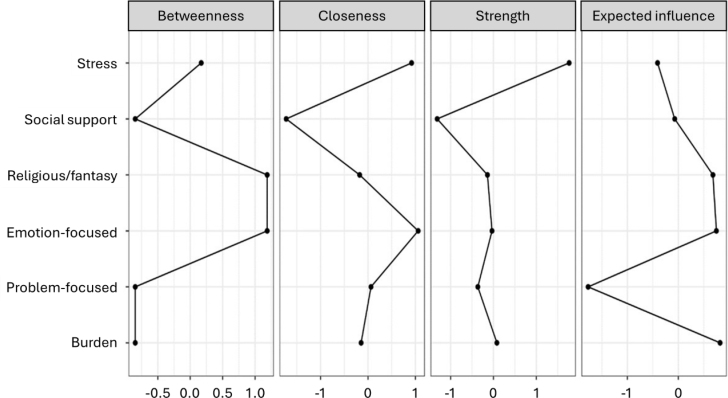
Betweenness, closeness, strength of connections, and expected influence of the network variables. Brazil, 2023.

Analyzing the network centrality indices (Figure 2), it was observed that emotion-focused coping and religious/fantasy-based coping presented the highest betweenness values, identifying them as the most influential nodes within the network. Perceived stress and emotion-focused coping exhibited the highest closeness centrality, with stress emerging as the network's most strongly connected variable.

Regarding expected influence, caregiver burden demonstrated the highest value, followed by emotion-focused coping and religious/fantasy-based coping. These findings suggest that activating this network depends primarily on these three nodes, which play a key role in influencing the activation of other variables.

## DISCUSSION

The study found that caregivers of older adults with AD reported moderate stress and mild burden, influenced by their coping strategies. Problem-focused strategies were protective, while emotion-focused and religious/fantasy-based strategies were associated with higher stress and burden. Network analysis showed greater centrality of emotional and religious strategies in these interactions.

A study by Bakof et al.^24^ also identified moderate stress and mild burden levels among caregivers of older adults with AD, which can be explained by the physical, emotional, and social demands of caregiving, compounded by family and professional responsibilities. The lack of support networks contributes to chronic stress and emotional vulnerability^25,26^.

Problem-focused coping was negatively associated with stress and burden, aligning with Cunha et al.^27^, who describe it as linked to managing symptoms, medications, and care routines. This strategy — characterized by planning and practical resolution — enhances caregivers’ sense of control and access to concrete resources^28–30^. However, not all caregivers manage to adopt this approach. Emotional exhaustion and lack of dementia-specific knowledge may lead to social withdrawal and reliance on less adaptive strategies^31,32^.

Conversely, emotion-focused coping was associated with higher stress and burden, consistent with Sena et al.^33^. This style includes crying, venting, or resignation, often offering only short-term relief^34,35^. Although common, it tends to reinforce distress and undermine long-term resilience.

Religious practices and social support also showed positive associations with burden, though the latter was more subtle. While religiosity can provide hope, calm, and a sense of belonging^36–38^, it may also foster passivity and unrealistic expectations when used as a sole coping resource^39^. Excessive reliance on divine intervention can generate a false sense of relief and perpetuate emotional overload.

Network analysis showed that emotion-focused and religious/fantasy-based strategies had the greatest centrality in the dynamics of coping, stress, and burden. Emotional strategies may act as temporary outlets^40^, while religiosity can foster resilience through meaning and motivation^41^. However, when used exclusively, they may inhibit active responses and intensify the caregiver's burden^42^.

Problem-focused coping emerged as the most protective strategy, reinforcing the idea that active problem-solving enhances perceived control and self-efficacy, improving psychological well-being^20,43^. In contrast, emotion-focused coping — despite offering emotional relief — fails to resolve the ongoing challenges of AD caregiving^44^. Avoidance of stressors tends to accumulate emotional strain, making adaptation more difficult^33^.

Similarly, religious/fantasy-based coping, while emotionally comforting, may foster unrealistic expectations. Faith alone cannot meet the complex demands of caregiving for individuals with progressive cognitive decline^45^. Furthermore, spirituality does not eliminate distress related to death, loss, and uncertainty, which may persist or intensify even in religious contexts^46^.

This study has some methodological limitations. First, the use of a non-probabilistic convenience sample may have introduced selection bias, as caregivers with internet access and greater interest in the topic may have been more likely to participate, which restricts the generalizability of the findings. Second, the exclusive use of self-report questionnaires raises the possibility of recall bias, since participants might have had difficulty accurately remembering or reporting their experiences. In addition, self-reporting can also be influenced by social desirability bias, leading caregivers to present their behaviors or emotions in a more favorable way.

Finally, as this was a cross-sectional design with online recruitment, there was no follow-up of participants, which precludes analysis of temporal changes and makes it impossible to assess loss to follow-up. Despite these limitations, the geographic diversity and the sample size provide a solid basis for descriptive analyses. Future research should consider probabilistic sampling, longitudinal designs, and mixed-methods approaches to deepen understanding of caregiver experiences and coping dynamics.

In conclusion, this study concluded that problem-focused coping was associated with lower levels of stress and burden, reinforcing its protective role. In contrast, emotion-focused and religious/fantasy-based coping strategies were linked to greater stress and burden, with network analysis confirming their central influence. These results emphasize the importance of interventions aimed at strengthening adaptive, problem-solving strategies among caregivers of older adults with AD.

## Data Availability

The datasets generated and/or analyzed during the current study are available from the corresponding author upon reasonable request.
